# Exploring the Dimensional Structure of Bullying Victimization Among Primary and Lower-Secondary School Students: Is One Factor Enough, or Do We Need More?

**DOI:** 10.3389/fpsyg.2019.00770

**Published:** 2019-04-04

**Authors:** Davide Marengo, Michele Settanni, Laura Elvira Prino, Roberto H. Parada, Claudio Longobardi

**Affiliations:** ^1^Department of Psychology, University of Turin, Turin, Italy; ^2^Department of Philosophy and Educational Science, University of Turin, Turin, Italy; ^3^School of Education, Western Sydney University, Sydney, NSW, Australia

**Keywords:** victimization, factor analysis, dimensional structure, bi-factor analysis, bullying

## Abstract

In adolescence, bullying victimization is typically represented in terms of a three-fold factor structure reflecting three components of verbal, physical, and social victimization. Recent studies have suggested the usefulness of alternativte models including both general and component-specific factors. In this study, we assessed the empirical and theoretical validity of an instrument assessing verbal, physical and social victimization using a set of alternative models of victimization: a unidimensional model, a three-factor model, and a bifactor model. Association between emerging factors and student variables were explored to establish theoretical fit of the models. Sample consisted of upper primary and lower secondary school students [*N* = 1311; 53% Male; Mean age (*SD*) = 10.73 (1.45)] and their teachers. The three factor and bifactor models showed good fit. In spite of acceptable fit, the unidimensional model showed lower empirical support when compared with the other models. The dimensions of the three-factor model showed similar associations with most student variables, while the bifactor showed more heterogeneous, and theoretically coherent associations. General victimization decreased with age and was positively related with externalizing and internalizing symptoms, student–teacher conflict and negative expectations. Verbal victimization showed increased prevalence among girls and older students. Physical victimization showed increased prevalence among boys and younger students, and positive associations with externalizing symptoms and student–teacher conflict. Social victimization was more frequent among girls, and positively related with internalizing symptoms and negative expectations toward teachers. These findings highlight the usefulness of modeling victimization using both general and form-specific dimensions for both assessment and theory-building purposes.

## Introduction

In-school bullying victimization refers to the continued, intentional aggression of a victim who is lower in power when compared to the aggressor, i.e., the bully (Olweus, 1996). Prevalence of bullying victimization among school-aged individuals is high: Cross-national studies show a prevalence rate ranging from to as low as 10% to as high as 30% of children and adolescents which reports being victim of some of form of bullying behavior at school (e.g., Eslea et al., 2004; Craig et al., 2009; Chester et al., 2015; for a review, see Zych et al., 2017).

When compared with uninvolved peers, bullies and their victims are both at greater risk of reporting increased emotional and behavioral problems (e.g., Smith et al., 2004; Schneider et al., 2012; Kowalski and Limber, 2013; Longobardi et al., 2018a; Marengo et al., 2018), as well as poorer in-school adjustment (e.g., increased student–teacher conflict, Marengo et al., 2018; lower academic engagement and achievement, Buhs et al., 2010; Rueger and Jenkins, 2014). Students who experience bullying victimization are at greater risk of developing long-lasting mental health problems, substance addiction (for a review, see Moore et al., 2017), as well as to experience social exclusion (Nansel et al., 2001; Hanish and Guerra, 2002; Delfabbro et al., 2006).

### The Components of Bullying Victimization

In literature, in-school bullying victimization is typically described as comprising three main components, each mirroring different forms of bullying – namely verbal, physical, and social/relational bullying (Crick et al., 2001; Cornell and Bandyopadhyay, 2010; Marsh et al., 2011; Harris et al., 2018). Students’ exposure to verbal (e.g., name calling, hurtful joking, and teasing) and physical (e.g., being hit, or being thrown things, or physically threatened) bullying behaviors is typically considered a direct form of victimization, while social/relational victimization is considered an indirect form of victimization which is aimed at damaging the social status of the victims (e.g., isolation, and exclusion from activities by peers, e.g., Arseneault et al., 2010). During childhood and adolescence, frequency of involvement in direct and indirect forms of victimization are often strongly correlated, meaning that typically victims tend to be exposed of both direct and indirect bullying behaviors (Casper and Card, 2017). Still, studies show the existence of differential relations of each form of victimization with students’ characteristics and psychosocial outcomes, supporting the importance of conceptualizing them as separate constructs.

First, in light of the heterotypic perspective on the development of bullying behaviors, the relative prevalence of different forms of bullying behaviors is expected to be different across age groups (Björkqvist et al., 1992, 1994; Underwood et al., 2009). According to this view, physical bullying should be more prevalent during middle childhood, while frequency of verbal and social/relational bullying should increase during late childhood and adolescence. Findings in support of this model have been mixed (e.g., Bettencourt et al., 2013; Longobardi et al., 2019), and seem to indicate that this transitional model appear to be more common among girls, in that they are more likely to show a form-specific developmental trend characterized by a transition toward lower physical and higher relational bullying over time (Card et al., 2008; Ettekal and Ladd, 2017). In turn, victimized boys tend to report high levels of involvement in all components of bullying victimization, thus supporting a general lack of independence of the different forms of victimization among boys (Ettekal and Ladd, 2017). Still, findings tend to indicate that physical victimization is generally more frequent among boys (Card et al., 2008), while relational victimization has been reported to be more frequent among females (Lee, 2009).

Studies also shows that different forms of victimization tend to show differential effects on students’ likelihood of reporting increased internalizing and externalizing symptoms. Although exposure to each form of victimization has been found to increase both kind of symptoms (Eastman et al., 2018), meta-analytic results suggest that exposure to social/relation victimization is more strongly associated with the risk of increased internalizing symptoms, while direct victimization is more strongly associated with externalizing symptoms (e.g., for a review and meta-analysis, Casper and Card, 2017). According to this view, students that are exposed to direct forms victimization are more likely to respond by direct forms of aggression; in turn, because of the subtleness of social/relational victimization behaviors and the difficulty of identifying specific perpetrators, direct confrontation tend not to be successful, leading the victims to internalize the negative experience.

Findings concerning the specific association between student–teacher relationship quality and students’ involvement in different forms of bullying victimization are scant. Overall, literature have shown that victimized students are more likely to show low-quality relationship with their teachers (Elledge et al., 2016) and report lower teacher support and safety in school (Raskauskas et al., 2010; Berkowitz and Benbenishty, 2012). In particular, lack of perceived support in school, increased student–teacher conflict, as well as excessive student–teacher dependency, have been shown to be linked to an increased risk of both direct and indirect forms of victimization (Hughes et al., 1999; Ostrov and Crick, 2007; Troop-Gordon and Kopp, 2011; Herráiz and Gutiérrez, 2016; Longobardi et al., 2016). However, it can be hypothesized that since teachers tend to be more lenient in responding to social/relational victimization than physical victimization (Xie et al., 2002; Bauman and Del Rio, 2006), and due to the covert nature of social/relational victimization (Crick and Grotpeter, 1995), low-quality student–teacher relationships may put students at increased risk of social/relation victimization. Indeed, findings indicate that students involved in relational victimization tend to report increased concerns about their safety in school (Elsaesser et al., 2013).

### Alternative Models of Bullying Victimization

Findings in support of the three-factor structure of bullying behaviors distinguishing between verbal, physical, and social/relational bullying are mixed (e.g., Björkqvist et al., 1992; Salmivalli et al., 2000; Crick et al., 2001). The few existing studies in support of the three-factor model highlighted the difficulty of modeling bulling and victimization data assuming independence of the verbal, physical and social components, thus have generally allowed the verbal, physical, and social factors to correlate (e.g., Marsh et al., 2004b, 2011; Unpublished doctoral dissertation; Finger et al., 2008; Harris et al., 2018). One of the limits of three-factor models allowing inter-factor correlations is represented by the high correlations found among the three different forms of bullying victimization. These findings suggests the opportunity to consider bullying victimization as a unidimensional construct. The presence of a general bullying victimization dimension would suggest the need for researchers to consider bullying victimization as a global experience: in this view, students differentiate among each other on the degree of direct exposure to bullying behaviors, and there is no need to distinguish among different forms of victimization. This approach is supported by empirical studies that developed and validated unidimensional scales including items referring to verbal, physical, and relational/social victimization (e.g., Kyriakides et al., 2006; Cheng et al., 2011; Shaw et al., 2013). Other studies have discussed the use of alternative models of victimization accounting for the strong associations existing between different forms of victimization by introducing a global victimization dimension along with a set of specific verbal, physical, and social dimensions (e.g., Finger et al., 2008; Harris et al., 2018). Among these alternative models, the bifactor model is the most promising as it allows to overcome some problems related to the high correlations found in the three-factor model between the verbal, physical and social victimization components. Using the bifactor model, it is possible to produce uncorrelated general and domain-specific factors (Reise et al., 2013), an approach that has both theoretical and analytical advantages over correlated-factors models. In particular, it allows for a clearer view on the specific associations between external variables (e.g., student psychological and behavioral outcomes) and the general and domain-specific dimensions, and helps reducing potential multicollinearity problems in analytical models (for examples of empirical applications in the educational context, see Betts et al., 2011; Wiesner and Schanding, 2013; Longobardi et al., 2018b).

### The Present Study

Firstly, our study aims to find out which model provide the best empirical fit to a self-report measure of bullying victimization among students’ attending upper primary and lower secondary schools. In light of previous literature, we compare the fit of three alternative models: (1) a model assuming victimization can be represented as a unidimensional construct, (2) a multidimensional model representing victimization as three correlated dimensions each referring to verbal, physical, and social victimization and (3) a final model in which victimization is represented in terms of a general victimization dimension along with three residual dimensions referring to verbal, physical, and social victimization. Coherent with this view, the aim of our second study is to examine the theoretical fit of competing models by examining and comparing the association emerging between the models’ dimensions and a set of student variables with known theoretical association with different forms of bullying victimization, i.e., students’ gender and age, internalizing and externalizing symptoms, and student–teacher relationship quality variables.

## Materials and Methods

### Sample

Sample consisted of 1311 students attending grade 3–8 in 8 upper-primary schools [51 classrooms, *N* = 807, 46.3% female Mean Age (*SD*) = 9.83 (0.65)] and 4 lower-secondary schools [32 classrooms *N* = 504, 48.6% female, Mean Age (*SD*) = 12.16 (1.20)] from Northern Italy. These demographic characteristics are representative of the Italian school system (Organization for Economic Co-operation and Development [OECD], 2011).

We also recruited students’ main teachers [i.e., teacher who spend the highest amount of weekly lesson time in the classroom, *N* = 83, 79 females, Mean Age (*SD*) = 50.79 (7.08), all Italian teachers]. It is worthy to note that in the Italian school system, main teachers typically teach to the same group of students from grade 1–5 (primary school), and from grade 6–8 (lower secondary school). That is, students only change teachers and classroom peers when they progress to the next school level (e.g., when transition from primary to lower-secondary school).

Data collection was performed by administering self-report questionnaires to both participating students and their main teacher. Students were asked to report about involvement in bullying victimization and student–teacher relationship quality with their main teacher, while teachers provided ratings for their students’ internalizing and externalizing symptoms. Prior to data collection, we obtained written and informed consent from all students and their parents. Prevalent teachers from the participating classrooms were also asked to sign an informed consent to take part in the study. In compliance with the ethical code of the Italian Association for Psychology (AIP), all participants were assured about of data confidentiality and informed of the nature and objective of the study, and that participation in the study was voluntary, i.e., that they could refuse to participate and withdraw from the study at any time. The study was approved by the IRB of the University (protocol no. 118643).

### Instruments

#### Bullying Victimization

Students’ involvement in bullying victimization was assessed using an adaption to the Italian context of the victimization sub-scales of the Adolescent Peer Relations Instrument (APRI, Parada, 2000). The APRI is psychometrically validated instrument that can be used to assess involvement in bullying behaviors as bully and victims. For the purpose of this study, we administered the victimization section of APRI instrument, which consists of 18 items (see Table 2) assessing three types of bullying victimization, namely physical (six items, α = 0.83), verbal (six items, α = 0.86), social victimization (six items, α = 0.81). The items are prefaced by a stem sentence asking students how many times various victimizing behaviors have happened to them for which they respond using a six-point Likert response scale (1 = Never, 2 = Sometimes, 3 = Once or twice a month, 4 = Once a week, 5 = Several times a week, 6 = Every day). Responses closer to 1 represented small amounts of being bullied, whereas scores closer to 6 represented frequent amounts of involvement in bullying victimization.

#### Students’ Internalizing and Externalizing Symptoms

Students’ internalizing and externalizing symptoms were assessed by administering a teacher-report instrument, Italian version of the Strength and Difficulties Questionnaire (SDQ; Goodman, 1997; Tobia et al., 2011). Teachers provided a rating of students’ symptoms by answering 25 items that refer to the positive or negative traits of the child’s behavior in class. The items are evaluated on a three-point Likert scale (i.e., Not True, Partially True, and Absolutely True), and asses five dimensions of children’s emotional and behavior characteristics: Emotional problems (e.g., “Often unhappy, downhearted”), Conduct problems (e.g., “: Often fights with other children”), Hyperactivity/inattention (e.g., “Easily distracted, concentration wanders”), Peer relationship problems (e.g., “Has at least one good friend”. reversed), and Prosocial behavior (e.g., “Considerate of other people’s feelings”).

As suggested by Goodman et al. (2010), for the purpose of this study items were combined to create two scores reflecting students’ internalizing symptoms (sum of emotional symptoms and problematic relationships with peers, α = 0.78), and externalizing symptoms (sum of conduct problems and hyperactivity/inattention symptoms, α = 0.88). Finally, we also computed the SDQ total score, which provides a measure of both internalizing and externalizing symptoms (sum of emotional symptoms, problematic relationships with peers, conduct problems and hyperactivity/inattention symptoms, α = 0.89).

#### Student–Teacher Relationship Quality

Students’ perceptions of student–teacher relationship quality was assessed using the Student Perception of Affective Relationship with Teacher Scale (SPARTS; Koomen and Jellesma, 2015). The SPARTS scale consists of 25 items investigating three dimension of student–teacher relationship, namely Closeness, Conflict, and Negative Expectations. The Closeness scale (8 items, α = 0.82) assesses the students’ positive feelings toward and reliance on their teacher (e.g., “I feel most at ease when my teacher is near”). Conflict (10 items, α = 0.79) refers to students’ perception of the extent of negative behavior, and attitudes experienced with their teacher (e.g., “I guess my teacher gets tired of me in class”). Negative expectations scale (7 items, 0.62) measures the lack of confidence in teacher’s responsiveness and availability, (e.g., “I wish my teacher knew me better”). The 25 statements are answered using 5-point response scale, ranging from 1 (“no, that is not true”), to 5 (“yes, that is true”).

### Analysis Strategy

**FIGURE 1:**
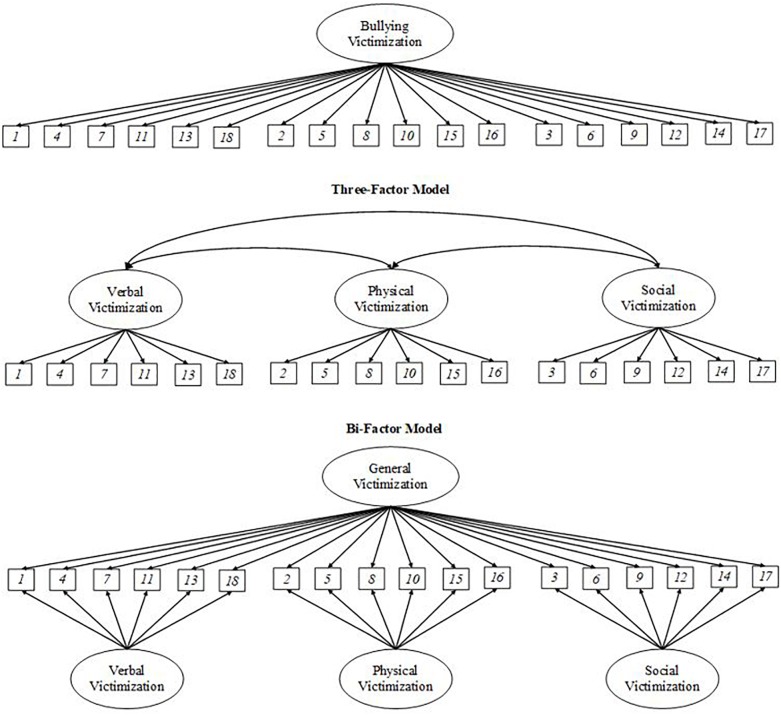
Diagrams for the one-factor, three-factor, and bifactor models of bullying victimization.

First, we compute descriptive statistics for study measures. Next, we use confirmatory factor analysis (CFA) to examine the fit of alternative models of victimization, namely a one-factor model, a three-factor model with correlated factors, and a bifactor model. Diagrams for these models are shown in Figure 1. CFA analyses were performed with the MPLUS software, version 7.3 (Muthen and Muthen, 2014). Since our data consists of ordered categorical responses with non-normal distribution, analyses are performed using the diagonally weighted least squares (DWLS) estimator (also known as the mean- and variance-adjusted weighted least squares, WLSMV; Unpublished technical report), which does not assume normally distributed variables and provides the best option for modeling categorical or ordered data (Brown, 2006). Approximately 2% of student observations had partial missing data; given the limited amount of missing data, and the fact that missing observations complied with the missing completely at random (MCAR) assumption of the WLSMV estimator (Asparouhov and Muthén, 2010) for unbiased estimates, we choose not to impute data and performed CFA analyses while retaining observations with missing data.

To establish model fit of the models we compute the following available model fit statistics: the comparative fit index (CFI: Bentler, 1990) and the Tucker–Lewis Index (TLI: Tucker and Lewis, 1973) measures of incremental model fit, the root mean square of approximation (RMSEA). Based on commonly used thresholds for model fit statistics in structural equation modeling (Hu and Bentler, 1999; Marsh et al., 2004a) we consider values of CFI > 0.95, TLI > 0.95, and RMSEA < 0.05 as indication of good model fit, while CFI and TLI > 0.90, and RMSEA < 0.08, as indication of acceptable fit. To compare the fit of nested CFA models, we perform χ^2^ difference tests. In the context of the present study, the one-factor model is nested in both the other competing models, since it can be obtained by either constraining the three-factor model to have inter-factor correlation of 1, or by constraining the domain-specific loadings of the bifactor model to 0. Since χ^2^ difference tests cannot be used to compare non-nested models (i.e., three-factor and bi-factor models), the models were compared using information-based fit statistics, namely Akaike’s and Bayes Information Criteria (AIC, BIC). Furthermore, differences in AIC values (Δ*AIC*) between the bestfitting model and the other tested models were examined to compare the fit of the models, and evaluated based on the criteria by Burnham and Anderson (2004), according to whom Δ*AIC* values > 10 indicate a substantially lower empirical support for the worse fitting model. Since analyses performed with the DWLS estimator does not produce an estimate of log-likelihood, AIC and BIC were estimated by running a secondary analysis using the robust maximum likelihood (MLR) estimator.

For both the unidimensional model and the three-factor CFA model, reliability of modeled factors is estimated by computing the omega (ω) model-based reliability coefficient (McDonald, 1985; Bentler, 2009). With respect to bi-factor model, reliability of general and specific factors was studied by computing the following indicators, as suggested by Rodriguez et al. (2016): coefficients omega (ω) and coefficients omega hierarchical for both general (ω_h_) and specific factors (ω_hs_). In the context of bi-factor model, omega is an estimate of the reliability of the general and specific factors based on all sources of common variance (Rodriguez et al., 2016). Concerning omega hierarchical statistics, when computed on the general factor omega hierarchical informs about the percentage of variance in the (summed) total score which can be attributed to the individual differences on the general factor. When computed on specific factors (i.e., verbal physical and social victimization), omega hierarchical informs about the percentage of reliable systematic variance in (summed) subscale scores after partitioning out variability attributed to the general factor (Reise et al., 2013). For each reliability estimate, we also compute its respective 95% confidence interval using 10,000 bootstrap samples.

Next, in order to estimate the bias introduced in the parameters of the one-factor model by not considering the existence of the different victimization components as potential source of multidimensionality, we computed the Average Relative Parameter Bias index (ARPB, Rodriguez et al., 2016). Finally, we compute the Percentage of Uncontaminated Correlation (PUC, Bonifay et al., 2015) index, which represents the percentage of covariance terms that only reflect the variance of the general factor, and the Explained Common Variance (ECV) for the general factor, which provides an estimate of the proportion of common variance explained by the general factor. In order to establish the degree the variance of individual responses to each item is accounted by the general factor alone, i.e., in order to establish the degree of unidimensionality of the item set, for each item we compute the Explained Common Variance index (IECV, Stucky et al., 2013). Previous authors have recommended I-ECV values above 0.80 as indication of unidimensionality (Stucky et al., 2013; Stucky and Edelen, 2014). Combined, inspection of these indexes can provide further insight on the degree of multidimensionality in the data.

Finally, based on parameters estimated in the previous analytical step, we generate the factor scores for the dimension of models showing adequate empirical fit and compute their correlation with a set of student variables, namely students’ age and gender, psychological symptoms and student–teacher relationship quality.

## Results

### Model Fit

Descriptive statistics for all of the study variables are reported in Table 1. Standardized factor loadings as well as model fit information for the final competing factor models are shown in Table 2.

**Table 1:** Descriptive statistics for study measures (*N* = 1311).

	Mean	*SD*	Observed range
Bullying victimization			
Verbal	10.28	5.21	6–36
Physical	8.37	3.71	6–36
Social	9.26	4.34	6–36
Psychological symptoms			
Internalizing symptoms	2.69	3.00	0–18
Externalizing symptoms	3.38	3.96	0–20
Total score	6.07	6.16	0–33
Student–teacher relationship			
Conflict	17.30	6.76	10–47
Closeness	25.92	7.62	8–44
Negative expectations	14.85	5.21	7–35

**Table 2:** Item statistics: factor loadings for the one-factor, three-factor model, and the bi-factor model of bullying victimization, and I-ECV values.

	One-factor	Three-factor			Bi-factor				
		
Items	General	Verbal	Physical	Social	General	Verbal	Physical	Social	I-ECV
01 I was teased by students saying things to me	0.72	0.74			0.72	0.24			0.90
04 A student made rude remarks at me	0.77	0.79			0.77	0.25			0.90
07 Jokes were made up about me	0.74	0.76			0.76				1.00
11 Things were said about my looks I didn’t like	0.75	0.77			0.74	0.39			0.78
13 I was ridiculed by students saying things to me	0.83	0.86			0.86				1.00
18 I was called names I didn’t like	0.66	0.68			0.66	0.17			0.94
02 I was pushed or shoved	0.67		0.74		0.61		0.42		0.68
05 I was hit or kicked hard	0.71		0.79		0.63		0.52		0.60
08 Students crashed into me on purpose as they walked by	0.67		0.74		0.62		0.37		0.74
10 My property was damaged on purpose	0.61		0.67		0.58		0.27		0.82
15 Something was thrown at me to hit me	0.68		0.76		0.60		0.52		0.57
16 I was threatened to be physically hurt or harmed	0.70		0.78		0.65		0.41		0.71
03 A student wouldn’t be friends with me because other people didn’t like me	0.68			0.73	0.63			0.50	0.62
06 A student ignored me when they were with their friends	0.60			0.65	0.56			0.42	0.64
09 A student got their friends to turn against me	0.75			0.80	0.74			0.24	0.90
12 I wasn’t invited to a student’s place because other people didn’t like me	0.64			0.68	0.59			0.45	0.63
14 A student got students to start a rumor about me	0.75			0.81	0.78				1.00
17 I was left out of activities on purpose	0.62			0.66	0.59			0.36	0.73
Model fit									
χ^2^ (df)	1022.752 (135)	469.95 (132)			259.58 (120)				
CFI	0.94	0.98			0.99				
TLI	0.93	0.97			0.99				
RMSEA	0.07	0.04			0.03				
AIC	37481.66	37035.96			36902.13				
BIC	38046.04	37615.87			37680.40				

Based on chosen criteria for CFI and RMSEA statistics, both three-factor and the bi-factor model presented good model fit, while the one-factor model showed acceptable fit. However, when compared with the other models, the one-factor model showed significantly worse fit to the data than both the three-factor [χ^2^(3) = 209.70, *p* < 0.001] and bi-factor model [χ^2^(18) = 527.42, *p* < 0.001]. Concerning the three-factor model, as found in other studies (Finger et al., 2008; Marsh et al., 2011; Harris et al., 2018), the latent factors for the three victimization components were highly correlated, with correlations ranging from *r* = 0.73 to *r* = 0.89. With respect to the model comparison of non-nested models (i.e., bifactor and three-factor models), inspection of information-based fit statistics did not allow for a clear-cut decision on which model showed best fit to the data. Inspection of AIC values indicated the bifactor model as the one showing best fit to the data (ΔAIC = 130.08) based on suggested threshold (ΔAIC > 10) when compared to the three-factor model. In contrast, comparison of BIC values indicated the three-factor model as the best fitting model.

Concerning specifically the bifactor model, item loadings on the general victimization factor were in the range 0.56–0.87, while loadings for the domain-specific factors were considerably lower, with 8 of the 18 items presenting loadings below |0.30|. Further, two items of the verbal victimization component (i.e., Item 7: “Jokes were made up about me”; Item 13: “I was ridiculed by students saying things to me”) and one item on the social victimization component (Item 14: “A student got students to start a rumor about me”) showed loadings falling below significance. Thus, the bifactor model was estimated again by constraining non-significant loadings to zero. Again, model fit for this revised bifactor model (see Table 2) showed better fit than the one-factor model [χ^2^(df) = 510.94 (15), *p* < 0.001]; concerning the comparison of non-nested models (i.e., bifactor and three-factor models), the inspection of AIC statistic again showed the bifactor model as the best fitting model, while the BIC statistic provided support for the three-factor model.

As regards factor reliability, the omega coefficients computed for the one-factor model (ω = 0.94 [95% CI = 0.94, 0.95]) and three-factor model (Verbal victimization: ω = 0.90 [95% CI = 0.88, 0.91]; Physical victimization: ω = 0.88 [95% CI = 0.87, 0.90]; Social victimization: ω = 0.86 [95% CI = 0.85, 0.88]) all showed good or excellent reliability. Concerning the reliability of factors emerging from the revised bifactor model, omega coefficients computed for both the general and the specific factors were all adequate (General victimization: ω = 0.95 [95% CI = 0.95, 0.96]; Verbal victimization: ω = 0.85 [95% CI = 0.83, 0.87]; Physical victimization: ω = 0.88 [95% CI = 0.87, 0.90]; Social victimization: ω = 0.86 [95% CI = 0.84, 0.87]). Inspection of omega hierarchical coefficient for the general victimization factor (ω_h_ = 0.89 [95% CI = 0.87, 0.90]) showed the general dimension accounted for the most part of the variance in the total score for general victimization. In turn, the omega hierarchical computed for the domain-specific factors showed the percentage of reliable variance in subscale scores only due to specific factors (i.e., after partitioning out the variance due to general victimization factor) was relatively low, in particular concerning the verbal component of victimization (Verbal victimization: ω_hs_ = 0.10 [95% CI = 0.06, 0.14]; Physical victimization: ω_hs_ = 0.28 [95% CI = 0.23, 0.33]; Social victimization: ω_hs_ = 0.25 [95% CI = 0.20, 0.30]).

Finally, by comparing the results of the patterns of loading of the one-factor and the bifactor model, a series of indexes, namely the ARPB, PUC, ECV, and I-ECV, were computed and inspected so as to provide further insight on the degree of appropriateness of modeling data using the bifactor model instead of using the one-factor model. The ARPB value computed on the item set was 0.06, which, combined with values of the PUC and ECV for the general factor, respectively, of 0.80 and 0.79, indicates that the degree of bias introduced in the parameters of the one-factor model by not considering the different components of victimization as sources of multidimensionality is relatively low. In contrast, inspection of I-ECV values computed for each item (see Table 2) shows that more than half the item-set, and, respectively, one, five, and four of the items of the verbal, physical, and social domains of victimization, reveal a non-negligible degree of multidimensionality (I-ECV < 0.80).

Overall, results showed that the one-factor model, in spite of an acceptable model fit, had lower empirical support when compared with other competing models. Further, the comparison of model fit of the best fitting models, namely the three-factor and bifactor CFA models, did not provide clear support for one model over the other. Hence, further investigation based on theoretical interpretability was needed in order to determine the appropriateness of modeling bullying victimization using either the three-factor or the bifactor model.

### Associations With Student Variables

Table 3 presents the associations between factor score estimates computed based on results of previously estimated three-factor and bifactor models and theoretically associated student variables. Inspection of correlations for the three-factor model scores indicated a similar pattern of associations with student variables. Concerning students’ demographic variables, each of three component of bullying victimization showed a negative association with students’ age, while gender (i.e., being female) showed a negative correlation with physical victimization. Concerning the remaining student variables, each of the three factors showed similar significant positive correlations with both students’ internalizing and externalizing symptoms measures, and the total score for symptoms. They also showed similar positive correlations with the student–teacher conflict and negative expectation measures, while no correlation emerged with student–teacher closeness.

**Table 3:** Three-factor and bi-factor model: correlation between victimization factors and student variables.

	Three-factor			Bi-factor			
	Verbal	Physical	Social	General	Verbal	Physical	Social
Gender (Female = 1; Male = 0)	−0.03	−0.20^∗∗^	0.03	−0.05	0.08^∗∗^	−0.23^∗∗^	0.15^∗∗^
Age	−0.07^∗^	−0.18^∗∗^	−0.10^∗∗^	−0.11^∗∗^	0.14^∗∗^	−0.14^∗∗^	−0.04
Psychological symptoms							
Internalizing symptoms	0.10^∗∗^	0.13^∗∗^	0.16^∗∗^	0.14^∗∗^	−0.03	0.06	0.10^∗∗^
Externalizing symptoms	0.12^∗∗^	0.20^∗∗^	0.16^∗∗^	0.16^∗∗^	−0.05	0.13^∗∗^	0.03
Total score	0.14^∗∗^	0.19^∗∗^	0.17^∗∗^	0.17^∗∗^	−0.05	0.11^∗∗^	0.07^∗^
Student–teacher relationship quality							
Conflict	0.27^∗∗^	0.34^∗∗^	0.24^∗∗^	0.31^∗∗^	0.01	0.20^∗∗^	0.00
Closeness	−0.02	−0.02	−0.04	−0.03	−0.02	0.00	−0.03
Negative expectations	0.29^∗^	0.25^∗∗^	0.32^∗∗^	0.32^∗∗^	0.04	0.06	0.13^∗∗^

*^∗^p < 0.05, ^∗∗^p < 0.01.*

Concerning the factor score estimates computed based on the revised bifactor model, the patterns of correlations computed between the general and specific victimization factors, and student variables were decidedly more heterogeneous. Concerning the general victimization factor, the emerging association were similar to those emerged when examining those computed for the three-factor scores. The general victimization score was negatively correlated with age, and showed a positive association with both students’ internalizing and externalizing symptom scores, as well as with the total score for symptoms. It also showed a positive correlation with student–teacher conflict and negative expectations toward the teacher.

Concerning the three specific victimization factors emerging from the bifactor model and their association with demographic variables, we found age was positively correlated with verbal bullying victimization and negatively correlated with physical victimization, while gender (being female) was positively correlated with verbal and social victimization, and negative correlated with physical victimization. Concerning students’ psychological symptoms, externalizing symptoms were positively associated with physical bullying but not with the other victimization components, while internalizing symptoms showed significant positive correlation with the social victimization component. Both physical and relational victimization also showed a positive correlation with the total score for symptoms. Finally, we found physical victimization was positively correlated with student–teacher conflict, while social victimization showed a positive association with students’ negative expectations toward teachers.

## Discussion

The present study aimed at establishing and comparing the empirical and theoretical fit of three alternative models of victimization, namely a unidimensional model, a model including three correlated dimensions of verbal, physical, and social victimization, and finally, a bifactor model including a general victimization dimension and three auxiliary dimensions reflecting the verbal, physical, and social components of victimization. In line with previous findings (Harris et al., 2018), results of factor analyses showed both the three-dimensional and bifactor model showed good empirical fit to the data. In turn, the unidimensional model showed acceptable fit but revealed lower empirical support when compared with the other models. For this reason, subsequent analyses focused solely on examining the theoretical fit of the three-dimensional and bifactor models, which we investigated by examining associations between the modeled dimensions and a set of student variables with known association with bullying victimization, namely students’ age and gender, psychological symptoms and student–teacher relationship quality.

As showed by previous studies (Marsh et al., 2004b, 2011; Parada, 2006; Finger et al., 2008; Harris et al., 2018), correlation between the verbal, physical, and social dimensions of the three-factor model were all quite high, supporting the usefulness of including a general victimization dimension in the model. Concerning the bifactor model, some of the items showed non-significant loadings on the specific verbal factor, namely items referring to being made jokes and being ridiculed by peers, and on the social factor, i.e., a student got other students starting a rumor. This led to a revision of the model in which these items were only allowed to load on the general victimization dimension; this final model showed similar fit to the data. Consistent with what emerged from the three-factor analysis, inspection of reliability of the dimensions emerging from the bifactor model revealed that most of the measurement variance was accounted for by a common general factor, while the variability associated solely with the specific dimensions was relatively low. This finding has important consequences for the use of the traditional three-factor structure of victimization, because it puts to question the appropriateness of using separate summed scores for the verbal, physical, and social victimization components (Reise et al., 2013).

Overall, inspection of the correlations between the factor scores computed using the three-factor and bifactor models and the set of student variables indicated the bifactor model provided a more differentiated and theoretically coherent representation of bullying victimization when compared with the three-dimensional model. In particular, the dimensions of the three-dimensional model showed remarkably similar patterns of associations with student characteristics, suggesting a general lack of distinctiveness among the three dimensions computed using the three-factor model: all forms of victimization showed a positive association with internalizing and externalizing symptoms, as well as student–teacher conflict and students’ negative expectations toward their teacher support. Consistently, the same pattern of associations emerged when inspecting correlations computed for the general victimization dimension of the bifactor model. Overall, findings were in line with the existence of a common association between different forms of victimization and increase in both internalizing and externalizing symptoms (e.g., Eastman et al., 2018), as well as their association with poorer student–teacher relationships (e.g., Troop-Gordon and Kopp, 2011; Herráiz and Gutiérrez, 2016), during childhood and early adolescence.

The inspection of correlations for the domain-specific factors of the bifactor model showed a more heterogeneous pattern of associations. Association with demographic variables was mostly coherent with theoretical expectations concerning the presence of difference in the prevalence of different forms of victimization across age and gender groups. Concerning age, we found a positive correlation with verbal victimization and a negative correlation with physical victimization; these results are in line with literature suggesting a decline in physical victimization, and a shift toward non-physical forms of victimization, as children grow older (e.g., Björkqvist et al., 1992, 1994; Underwood et al., 2009). However, contrary to expectations, we did not find positive association between age and relational bullying, which may be partially due the lack of older adolescents in the sample of the study. Concerning gender, results were coherent with findings indicating an increased prevalence of physical victimization among boys, and of relational victimization among girls (Card et al., 2008; Lee, 2009; Longobardi et al., 2017).

Findings emerging from the inspection of correlations with students’ psychological symptoms and student-relationship quality showed also a more differentiated pattern when compared with those emerging from the three-factor model. As regards students’ psychological symptoms, results were in a line with meta-analytic results indicating that overt forms of victimization, such as physical victimization, tend to be associated with increased externalizing symptoms, while covert forms of victimization, such as social/relational victimization, might put students at increased risk of developing internalizing symptoms (Casper and Card, 2017). Concerning the association with student-relationship quality, we found that physical victimization was positively related with student–teacher conflict, while social victimization showed a positive association with negative expectations toward teachers’ support. Overall, the association between physical victimization and heightened student–teacher conflict may be interpretable in light of a possible negative influence of victims’ externalizing behaviors on student–teacher relationship (e.g., Ostrov and Crick, 2007; Troop-Gordon and Kopp, 2011). In turn, the specific association between social victimization and negative expectations toward teachers appear to be coherent with findings evidencing the perception of lack of support from teachers among victims of indirect forms of victimization (Yeung and Leadbeater, 2010; Lucas-Molina et al., 2015).

Overall, similar to other studies investigating the empirical and theoretical fit of bifactor models in modeling multidimensional data including correlated latent dimensions (e.g., Simms et al., 2008; Martel et al., 2011; Hindman et al., 2016; Longobardi et al., 2018b), in the present study the use of the bifactor model proved beneficial in elucidating the existence of both general and domain-specific dimensions of victimization, and in providing a clearer view on the association between each dimension and theoretically associated external criteria. Still, a word of caution should be spent as regards the interpretation of bifactor scores for domain-specific dimensions when estimated reliability is low, as this is expected to compromise replicability of results across different samples. To counteract this problem and improve reliability of domain-specific scores, researchers should consider increasing the length of administered questionnaires by including more items assessing the domain-specific dimensions.

This research is not without limitation. First of all, use of a non-random, convenience sample of Italian students, which limits the generalizability of results to larger populations. On the other side, because of the use of a relatively large sample, our results seem to be very robust. Second, we could not retrieve information about other important student variables which may have helped further examined the construct validity of victimization dimensions, such as information about academic performance, as well as sociometric data. Finally, we did not analyze data concerning students’ involvement in bullying behaviors. This would have allowed us to test to compare the adequacy of the bifactor model for both bullying and victimization behaviors.

## Conclusion

Results from the present study highlight the usefulness of modeling victimization using both a general victimization dimension and a set of specific dimensions representing verbal, physical, and social forms of victimization. The alternative representations of victimization (bi-factor and three factor model) are similar in terms of fit, but there may be advantages of one or the other in particular situations. The three-factor model can produce scores for each component of victimization that while strongly correlated, have high score reliability, a characteristic that supports their use for assessment purposes (e.g., identification of students at risk of one or more forms of victimization). Using the bifactor model it is possible to obtain a highly reliable, unidimensional score representing the degree of student’s involvement in all forms of bullying victimization, and a set of indicators for the specific forms of victimization that show lower reliability but do not suffer from the presence of collinearity typical of scores emerging from the three-factor model. In doing this, it allows for a clearer picture of the studied constructs than that obtained using traditional multidimensional approaches and can highlight limits and possible drawbacks related to the use of traditional scoring procedures. This represent an important advantage with non-trivial consequences on the ability to evidence the existence of specific association between students’ psychosocial outcomes and their exposure to different forms of bullying victimization.

## Ethics Statement

School principals gave their consent for the participation of both students and teachers in our study. Individual informed consent to take part in the research was also collected from teachers, students, and their parents. Data confidentiality was assured and participation to the study was voluntary. The study was approved by the IRB of the University of Turin.

## Author Contributions

DM worked on the introduction, analyses, and discussion. MS worked on the analyses and discussion. CL worked on the data collection, introduction, and discussion. LP and RP worked on the introduction and discussion.

## Conflict of Interest Statement

The authors declare that the research was conducted in the absence of any commercial or financial relationships that could be construed as a potential conflict of interest.
